# The C-Terminal Domain of the *Bacillus thuringiensis* Cry4Ba Mosquito-Specific Toxin Serves as a Potential Membrane Anchor

**DOI:** 10.3390/toxins11020062

**Published:** 2019-01-23

**Authors:** Anon Thammasittirong, Chompounoot Imtong, Wilaiwan Sriwimol, Somsri Sakdee, Chanan Angsuthanasombat

**Affiliations:** 1 Microbial Biotechnology Unit, Department of Microbiology, Faculty of Liberal Arts and Science, Kasetsart University, Nakornpathom 73140, Thailand; faasant@ku.ac.th; 2 Division of Biology, Department of Science, Faculty of Science and Technology, Prince of Songkla University, Pattani 94000, Thailand; chompou_ron@hotmail.com; 3 Department of Pathology, Faculty of Medicine, Prince of Songkla University, Songkla 90100, Thailand; wilaiwan.sr@psu.ac.th; 4 Bacterial Toxin Research Innovation Cluster (BRIC), Institute of Molecular Biosciences, Mahidol University, Salaya Campus, Nakornpathom 73170, Thailand; sakdees@gmail.com; 5 Laboratory of Synthetic Biophysics and Chemical Biology, Biophysics Institute for Research and Development (BIRD), Chiang Mai 50130, Thailand

**Keywords:** Cry4Ba toxin, C-terminal domain, membrane anchor, pore formation, proteinase K, β-sheet structure

## Abstract

Although the C-terminal domain (DIII) of three-domain Cry insecticidal toxins from *Bacillus thuringiensis* has been implicated in various biological functions, its exact role still remains to be elucidated. Here, the 21-kDa isolated DIII fragment of the 65-kDa Cry4Ba mosquito-specific toxin was analyzed for its binding characteristics toward lipid-bilayer membranes. When the highly-purified Cry4Ba-DIII protein was structurally verified by attenuated total reflection Fourier transform infrared (ATR-FTIR) spectroscopy, it revealed the presence of a distinct β-sheet structure, corresponding to its structure embodied in the Cry4Ba crystal structure. Binding analysis via surface plasmon resonance (SPR) spectroscopy revealed that the 21-kDa Cry4Ba-DIII truncate displayed tight binding to immobilized liposome membranes in a two-step manner, exhibiting a dissociation rate constant (*k*_d_) comparable to the 65-kDa full-length toxin. Also similar to the Cry4Ba full-length toxin, its isolated DIII truncate was able to anchor a part of its molecule into the immobilized membrane as the SPR signal was still detected after prolonged treatment with proteinase K. However, unlike the full-length active toxin, the DIII truncate was unable to induce membrane permeability of calcein-loaded liposomes or ion-channel formation in planar lipid bilayers. Together, our present data have disclosed a pivotal role of C-terminal DIII in serving as a membrane anchor rather than a pore-forming moiety of the Cry4Ba mosquito-active toxin, highlighting its potential mechanistic contribution to the interaction of the full-length toxin with lipid membranes in mediating toxicity.

## 1. Introduction

**Figure 1:**
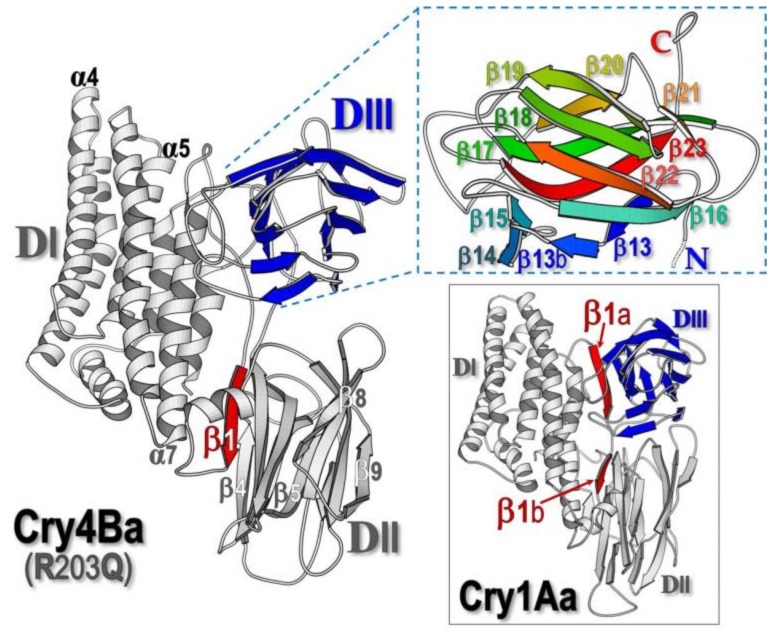
Ribbon presentation of the 65-kDa three-domain toxin structures, Cry4Ba-R203Q [9] (*left pane*) and Cry1Aa (*lower-right panel*) [7], of which DIII displays the inner and outer sheets (*blue shading*) and the location of β_1_ (*red shading*). *Upper-right panel*, ribbon representation of a different view of Cry4Ba-DIII by facing the inner sheet upwards, with the rainbow spectrum shading from N-terminus to C-terminus.

Strains of *Bacillus thuringiensis* (*Bt*), a Gram-positive bacterium producing insecticidal proteins known as crystal (Cry) and/or cytolytic (Cyt) δ-endotoxins [1], have been widely used as highly specific and safe bio-insecticides for the control of agricultural pests and human disease vectors [2,3]. Of particular interest, the Cry4Ba mosquito-active toxin produced from *Bt* subsp. *israelensis* is specifically toxic to the larvae of *Aedes* and *Anopheles* spp., vectors of important tropical infectious diseases of dengue hemorrhagic fever and malaria, respectively [3,4,5]. Upon ingestion by susceptible insect larvae, Cry toxins, which are initially produced as protoxin inclusions, are solubilized in the larval midgut lumen (highly alkaline for dipteran and lepidopteran larvae) prior to proteolytic activation by gut proteases to yield active toxins of ~65 kDa [1,5]. The activated Cry toxins display a wedge-shaped architecture with three structurally distinctive domains [6,7,8,9]: an N-terminal α-helical bundle (DI), a β-sheet prism (DII) and a C-terminal β-sheet sandwich (DIII) (see Figure 1). In particular, DI and DII of numerous Cry toxins have been evidently demonstrated to be responsible for membrane-inserted pore formation and target receptor recognition, respectively [10,11].

In our previous studies, the functional importance of both DI and DII of the two closely related mosquito-specific toxins (i.e., Cry4Aa and Cry4Ba) was intensively investigated [12,13,14,15,16,17,18]. Of particular significance, we have disclosed the intrinsic stability toward the Pro-rich cluster (Pro^193^Pro^194^_Pro^196^) which is present exclusively in the long loop linking two pore-lining helices (α_4_ and α_5_) within Cry4Aa-DI [13]. We have also shown that two highly conserved aromatic residues (i.e., Tyr^249^ and Phe^264^) in α_7_ of Cry4Ba-DI could play an important role in toxin-membrane interactions, believably required for lipid-induced conformational changes prior to an efficient insertion of the transmembrane α_4_-loop-α_5_ hairpin into the lipid membrane [14]. Moreover, the polarity of the α_4_-α_5_ loop residues—Asn^166^ was demonstrated to be crucial for ion permeation through the Cry4Ba-induced pore, likely to promote the toxin-pore opening [15]. For a functional role in receptor binding of DII, while most other investigators’ studies are restricted to merely three β-hairpin loops, i.e., β_2_-β_3_, β_6_-β_7_ and β_10_-β_11_ loops (originally assigned as loops 1, 2 and 3, respectively [7]), we have revealed that two other Cry4Ba-loops, i.e., β_4_-β_5_ and β_8_-β_9_ loops, are also important for receptor binding, and hence larvicidal activity [16]. More recently, we have shown that the stability of two receptor-binding hairpins (i.e., β_2_-β_3_ and β_4_-β_5_ within Cry4Ba-DII) contributed by hydrogen bonding between Thr^328^-Thr^369^ side-chains is important for toxin binding to Cyt2Aa2—an alternative receptor for the Cry4Ba toxin [17,18]. Furthermore, we have succeeded in identifying two different types of Cry4Ba receptors from *Aedes* mosquito larvae, i.e., glycosylphosphatidylinositol (GPI)-anchored alkaline phosphatase and GPI-anchored aminopeptidase N (APN) [19,20].

Unlike DI and DII of Cry toxins, the role of DIII remains ambiguous although it has been suggested to be involved in maintaining the structural integrity of the active toxin [7,21,22,23] or in determining binding specificity [24,25,26]. Recently, DIII was also suggested to participate in the interaction of Cry1Ie with the larval peritrophic membrane of *Ostrinia furnacalis* (Asian corn borer) [27]. In our previous study, the 21-kDa isolated DIII fragment from the Cry4Ba mosquito-active toxin was shown to be capable of binding along the apical microvilli of *A. aegypti* larval midgut, conceivably participating in toxin interactions with either lipid membranes or target protein receptors [28]. However, a precise description of such interactions would need further investigation. Here, we have further demonstrated the functional significance of C-terminal DIII of Cry4Ba for serving as a tight-binding lipid anchor, highlighting its potential contribution to toxin interactions with the bilayer membrane to mediate toxicity.

## 2. Results and Discussion

### 2.1. Structural Feature of the Isolated Cry4Ba-DIII Truncate Revealed by ATR-FTIR

**Figure 2:**
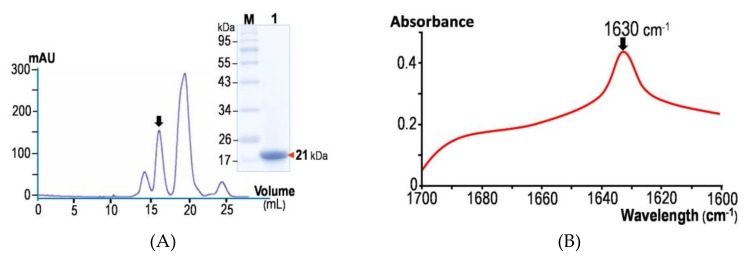
(**A**) Size-exclusion FPLC chromatogram of the isolated Cry4Ba-DIII protein. *Inset*, SDS-PAGE analysis (Coomassie brilliant blue-stained 12% gel) of a selected peak fraction (*arrow*) containing the 21-kDa purified DIII protein. M, molecular mass standards. (**B**) Non-polarized ATR-FTIR spectrum of the 21-kDa purified Cry4Ba-DIII protein from 1700 to 1600 cm^−1^. The arrow indicates the amide I peak at wavelength of 1630 cm^−1^.

The optimized preparation procedure established previously for the cloned Cry4Ba-DIII protein, which was over-expressed in *E. coli* as a soluble form [28], allowed us to obtain sufficient amounts of the purified DIII protein for subsequent characterization of its properties. Prior to binding studies, a high-purity Cry4Ba-DIII protein obtained by sequential ion-exchange and size-exclusion FPLC (fast protein liquid chromatography) (see Figure 2A, *inset*) was structurally verified by ATR-FTIR spectroscopy. As shown in Figure 2B, the amide I region (1700–1600 cm^−1^) of the non-polarized FTIR spectrum from the 21-kDa purified Cry4Ba-DIII truncate exhibited a prominent contribution with a maximum absorbance at 1630 cm^−1^ that is the signature of a β-sheet structure, thus signifying that the isolated Cry4Ba-DIII truncate adopts a native-folded β-structure, albeit detached from the N-terminal DI–DII portion (Figure 1). It should be noted that the 21-kDa Cry4Ba-DIII protein most likely exists as a monomer since its elution profile from the size-exclusion FPLC analysis revealed that the purified truncate was eluted as a single peak in line with the elution volume of the 23-kDa lysozyme protein marker (Figure 2A).

The structural feature of the isolated Cry4Ba-DIII truncate presented here is rather different from what has been reported elsewhere for both cloned Cry1Ab-DIII and Cry1Ac-DIII truncates which could be found only in the insoluble form when overproduced in *Escherichia coli* [29]. Dissimilarity in the cloning region for their DIII termini may perhaps contribute to such structural discrepancies between the isolated DIII truncates of Cry1A and Cry4Ba toxins. As can be inferred from the Cry1Aa crystal structure [8] that its DIII, which encompasses β_12_–β_23_ (residues 463 to 609), appears to directly interact with an extra β-strand (i.e., β_1a_, residues 254 to 264) which is highly conserved amongst Cry1A toxins. This particular feature may suggest an important role for β_1a_ in maintaining the structural integrity of DIII in the Cry1A toxins, but not in the Cry4Ba toxin (see Figure 1). Accordingly, the lack of this exclusive β-strand might result in improper folding of the isolated Cry1Ab- and Cry1Ac-DIII truncates, leading to the formation of an insoluble aggregate of mis-folded proteins. This notion has been supported by another recent study for overproduction of the 28-kDa His-tagged Cry1Ie fragment which was also found to be in the form of a mis-folded insoluble aggregate [27].

### 2.2. Membrane-Binding Characteristics of the Isolated Cry4Ba-DIII Truncate

**Figure 3:**
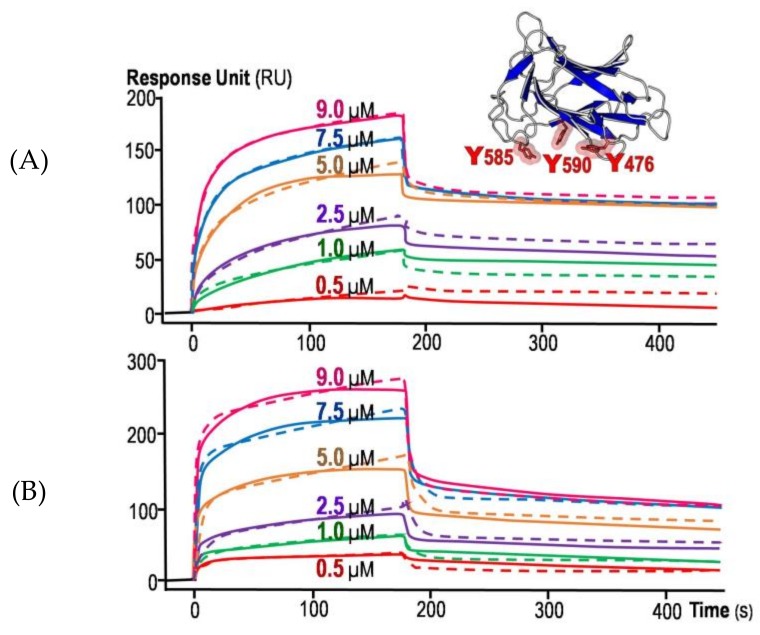
Membrane-binding analysis via SPR spectroscopy. Data analysis performed by global fitting of the sensorgrams from the 21-kDa isolated Cry4Ba-DIII protein (**A**) or its 65-kDa full-length toxin (**B**) with the two-stage reaction model. The experimental curves of each concentration (*solid line*) are shown alongside the fitted curves (*dashed line*). *Inset* in (**A**), ribbon representation of isolated Cry4Ba-DIII, illustrating a surface-exposed aromatic cluster (Tyr^476^, Tyr^585^ and Tyr^590^) that could serve as a membrane-binding region.

As previously demonstrated via immuno-histochemical assay, the cloned Cry4Ba-DIII fragment was able to bind to the midgut apical microvilli of the susceptible *A. aegypti* larvae [28]. In the present study, further attempts were made via more insightful approaches to reveal a binding target of the Cry4Ba-DIII truncate and hence its potential mechanistic role in toxicity. The binding capability of the 21-kDa Cry4Ba-DIII truncate to lipid-bilayer membrane was investigated in comparison with its full-length toxin to evaluate differences by real-time binding analysis via SPR spectroscopy. As shown in Figure 3, both truncated and full-length proteins showed signs of tight binding to the immobilized lipid bilayers as their complete dissociative events in SPR-sensorgrams were unachieved even if the dissociation phase was prolonged to 4 h, suggesting a degree of their membrane insertion. To further determine their association and dissociation rate constants, SPR sensorgrams were obtained with different concentrations of each ligand protein (see Figure 3). As the concentrations of the Cry4Ba-DIII truncate increased, its raised binding signals (expressed in resonance units, RU) were observed dose-dependently (Figure 3A). Likewise, similar dose-dependent SPR responses were also revealed for the full-length toxin (Figure 3B).

To further analyze their binding kinetics, the SPR-binding curves obtained with both the DIII truncate and its full-length protein were fitted to various models. The best-fit obtained was with a “two-stage reaction (conformation change)” model represented by the equation: *A* + *B*↔*AB*↔AB* which indicates further changes of the bound complex *AB* into AB*. This two-stage reaction model which, in terms of protein-lipid membrane interaction, would correspond to stage 1, a protein (*A*) binding to a lipid membrane (*B*) to give *AB*, followed by stage 2, the complex *AB* changing to AB* which cannot dissociate directly to *A* + *B* and which could correspond to insertion of the protein into the bilayer membrane. This notion is in good agreement with several previous studies that such a two-stage reaction model was successfully applied in SPR-sensorgram fitting for the case of a single-ligand molecule versus lipid-membrane surface [30,31,32].

Apparent rate constants of both ligand proteins obtained from this model are shown in Table 1. Accordingly, it is likely that the Cry4Ba-DIII truncate would initially bind to lipid bilayers at a slower rate (with ~3-fold lower *k*_a1_ value) than the full-length toxin, implying that other domains could be involved in the initial binding stage; in particular, DI (the pore-forming domain) of which a bundle of hydrophobic N-terminus regions was previously proposed to initiate the toxin binding to the receptor-free lipid membrane [33]. However, the *k*_a2_ value of the Cry4Ba-DIII protein was found to be ~4-fold higher than that of the full-length toxin, suggesting that this truncated protein would proceed more rapidly to the next binding stage (membrane-embedded stage). Conceivably, its smaller size would make the DIII truncate to step into the next stage more readily, possibly squeezing into the bilayers faster than the larger-size, full-length toxin. Interestingly, the dissociation rate constants (*k*_d1_ and *k*_d2_) of both Cry4Ba-DIII and full-length proteins were found roughly in the same order of magnitude, suggestive of their similar strength used for binding to the lipid bilayers.

As already noted in several studies, aromatic residues of various membrane-interacting proteins could play an important role in membrane interactions, depending on their location and side-chain orientation [32,34,35]. In particular, a solvent-exposed aromatic cluster of Equinatoxin II, a 20-kDa pore-forming toxin from the sea anemone *Actinia equine*, was shown to be the most important for membrane binding [32]. Herein, structure-sequence analysis of Cry4Ba-DIII revealed a surface-exposed aromatic cluster comprising Tyr^476^, Tyr^585^ and Tyr^590^ (see Figure 3A, *inset*). Despite the fact that these three Tyr residues appear to be marginally conserved among the Cry toxin family, their side-chain positions as highly exposed on one side of the DIII surface could allow them to be a potential membrane-binding cluster.

### 2.3. Cry4Ba-DIII Serves as a Membrane Anchor but not a Membrane-Perturbing or Pore-Forming Moiety

**Figure 4:**
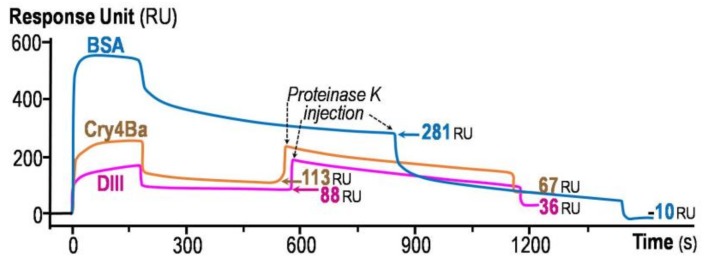
Real-time sensorgrams obtained from proteinase K protection assays of the isolated Cry4Ba-DIII protein (*pink*), its full-length toxin (*orange*) and BSA (*blue*) at the protein concentration of 9 µM. Injections of proteinase K to individual membrane-associated proteins are indicated by arrows. Horizontal arrows indicate resonance units (RU) values at the start and the end of proteinase K injection phase.

To determine if the lipid-associated Cry4Ba-DIII truncate is deeply embedded into the immobilized lipid bilayers or peripherally attached to the membrane surface, further SPR experiments were conducted via protease-digestion protection assays in comparison with its 65-kDa full-length toxin and the 66-kDa BSA (bovine serum albumin) control. As can be seen in Figure 4, SPR-binding sensorgrams of both Cry4Ba-DIII and full-length proteins were remarkably dissimilar to that of the BSA control, suggesting their differences in membrane association and dissociation. Throughout a prolonged period of 10-min incubation with proteinase K, the SPR signals of both Cry4Ba proteins started to decrease gradually and finally remained at levels notably higher than the basal value, while that of the BSA control appeared to decline more rapidly to the initial baseline (Figure 4). These results indicated that the immobilized bilayer membrane was able to protect both the DIII truncate and its full-length toxin, but not BSA, from the complete removal by proteinase K digestion, suggesting that some parts of these two Cry4Ba molecules are embedded in the lipid bilayers. Our observations are partly consistent with other previous studies using proteinase K protection assays on CryIA insertion into *Manduca sexta* larval BBMVs (brush-border membrane vesicles) since they showed that not only DIII but also DI and DII could be buried in such insect BBMVs, albeit to different extents, proposing an insertion of almost the entire toxin of ~60 kDa into the target insect membranes to mediate larvicidal activity [36].

**Figure 5:**
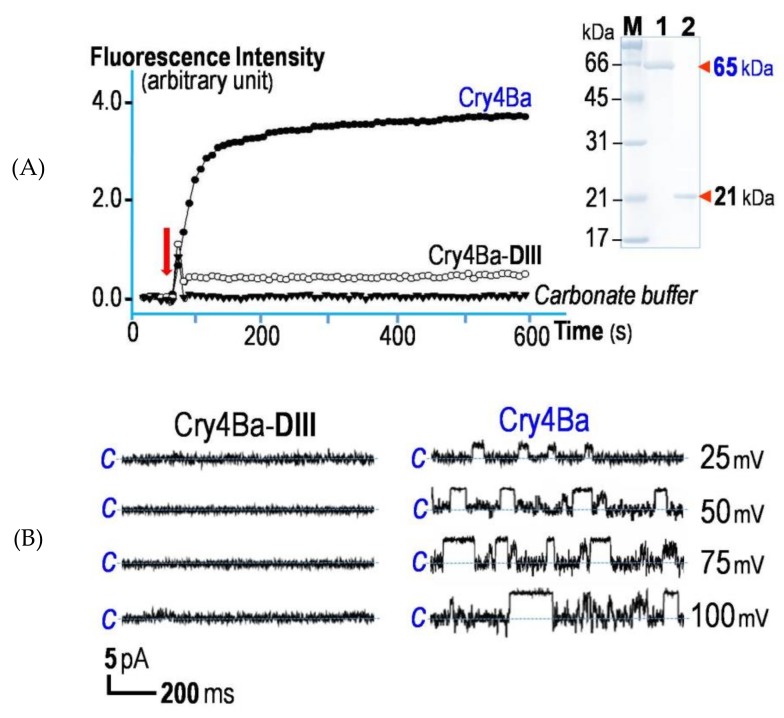
(**A**) Effects on permeability of calcein-loaded liposomes induced by Cry4Ba-DIII or its full-length toxin. Fluorescence intensity of the entrapped calcein upon release from LUVs continuously monitored as function of time after incubation with Cry4Ba-DIII (~30 µg/mL), its full-length toxin (~10 µg/mL) or the control buffer. *Inset*, SDS-PAGE analysis (Coomassie brilliant blue-stained 12% gel) of the 65-kDa purified Cry4Ba active toxin (*lane 1*) and the 21-kDa DIII truncate (*lane 2*). M, molecular mass standards. (**B**) Channel-forming characteristics of Cry4Ba-DIII and its full-length toxin formed in PLBs. Current traces, I (pA) *versus* time (s), recorded after reconstitution of purified Cry4Ba-DIII (*left panel*) and its full-length toxin (*right panel*) into *cis* chamber under a symmetrical condition (150 mM KCl, 10 mM Tris-HCl, pH 9.0 and 1 mM CaCl_2_). Applied voltages are indicated on the right side of each trace. Closed stage levels are denoted by the letter *c*. Each experiment was performed at least in duplicate.

Further attempts were made to examine if the isolated Cry4Ba-DIII truncate would be able to induce membrane permeability of calcein-loaded LUVs (large unilamellar vesicles) in comparison with its full-length active toxin. Using LUV-leakage fluorescence assays, the release of the encapsulated calcein by tested toxins was quantified as the relative increase in fluorescence de-quenching intensity. As demonstrated in Figure 5A, unlike with the full-length active toxin, the extent of entrapped calcein release when tested with Cry4Ba-DIII was still undetectable even after incubation over 10 min, suggesting that the isolated Cry4Ba-DIII truncate does not function as a membrane-perturbing unit. It is therefore possible that DIII was not deeply inserted into the lipid bilayers albeit the proteinase K protection results, suggesting an insertion of a part of the DIII molecule into the immobilized bilayers. Likewise, further functional characterization via PLBs (planar lipid bilayers) revealed that the isolated DIII truncate was incapable of forming ion channels in the bilayer membrane dissimilar to its full-length active toxin (Figure 5B). Thus, the PLB data corroborate the notion that isolated Cry4Ba-DIII cannot span the lipid membrane to form a leakage pore unlike the isolated DI-helical domain as previously characterized to be responsible for ion-leakage pore formation [37,38].

Taken together, our present data have provided evidence for a pivotal mechanistic role of Cry4Ba-DIII in membrane anchoring of the full-length active toxin rather than membrane perturbation or pore formation. However, a possible involvement of such a C-terminal β-sheet sandwich domain (DIII) in receptor binding still needs further investigation.

## 3. Materials and Methods

### 3.1. Preparation of the Cry4Ba Active Toxin

The 65-kDa Cry4Ba full-length active toxin was prepared from the 130-kDa Cry4Ba-R203Q mutant protoxin in which one trypsin-cleavage site at Arg^203^ was replaced with Gln, thus giving a 65-kDa activated toxin upon digestion with trypsin and retaining high larval toxicity as described elsewhere [9]. Toxin preparation was accomplished by digestion of the protoxin pre-solubilized in carbonate buffer (50 mM Na_2_CO_3_/NaHCO_3_, pH 9.0) with trypsin (*N*-tosyl-L-phenylalanine chloromethyl ketone-treated, Sigma-Aldrich, St. Louis, MO, USA) prior to purification by size-exclusion FPLC using Superose^®^ 12, HR10/30 (GE Healthcare Bio-Sciences, Uppsala, Sweden) as described elsewhere [39]. Finally, the purified toxin was analyzed by sodium dodecyl sulfate-(12% *w*/*v*) polyacrylamide gel electrophoresis (SDS-PAGE) and its concentration was determined by Bradford microassay (Bio-Rad, Hercules, CA, USA).

### 3.2. Expression and Purification of the Cry4Ba-DIII Truncate

Upon 4h-induction with IPTG (isopropyl β-D-1-thiogalactopyranoside), the cloned Cry4Ba-DIII truncate was over-expressed as a soluble form in *E. coli* strain JM109 under control of the *lac* promoter. The 21-kDa Cry4Ba-DIII protein was subsequently purified by ion-exchange and size-exclusion FPLC according to the procedure described previously [28]. After SDS-PAGE analysis, the concentration of the purified protein was determined by Bradford microassay.

### 3.3. Structural Verification of the Cry4Ba-DIII Truncate via ATR-FTIR Spectroscopy

The purified Cry4Ba-DIII truncate was dialyzed against deionized water and then applied onto a trapezoidal germanium internal reflection element (20 mm × 50 mm × 2 mm dimension, Graseby Specac Inc., Kent, UK). After extensively purging the instrument with N_2_ gas, ATR-FTIR spectra were collected on a Nicolet Nexus 470 spectrometer (Thermo Nicolet, Waltham, MA, USA) equipped with an MCT/A detector. The measurements were done with a 25-reflection ATR accessory and a wire grid polarizer (0.25-μm wire spacing, Graseby Specac Inc., Kent, UK). A total of 200 interferograms was recorded at 25 °C using either parallel (||) or perpendicular (⊥) polarized light at a resolution of 4.0 cm^−1^ prior to being processed with 1-point zero filling, Happ-Genzel apodization and automatic baseline correction. The secondary structure of the DIII protein was determined using the corrected ATR spectra which were derived from parallel (||) or perpendicular (⊥) polarized spectra, according to mathematical formula [1 (||) + 1.44 (⊥)] [40].

### 3.4. Membrane-Binding Assays of the Cry4Ba Proteins via SPR Spectroscopy

Membrane interactions of the Cry4Ba-DIII truncate as well as the 65-kDa Cry4Ba full-length toxin were determined with an SPR biosensor at 25 °C using the BIAcore-X system (Biacore AB, Uppsala, Sweden). The surface of the L1 sensor chip coated with carboxymethylated dextran was cleaned with 40 mM *n*-octyl-β-d-glucopyranoside (OG, Sigma-Aldrich, St. Louis, MO, USA). Then, a suspension of 0.5 mM lipid-membrane sample in the form of liposome (50-nm diameter) prepared from a lipid mixture (Avanti Polar Lipids Inc., Alabaster, AL, USA) of phosphatidylethanolamine (PE), phosphatidylcholine (PC) and cholesterol (Ch) (10:10:1, *w*/*w*/*w*) was injected onto the chip surface at flow rate of 2 μL/min for 30 min. The multi-lamellar structure of the lipid membrane was removed from the SPR surface with 0.01 N NaOH. After the binding to the sensor chip coated with lipophilic dextran, the immobilized liposomes would spontaneously fuse to form a large single-bilayer membrane as illustrated in Figure S1 (Supplementary materials). A typical sensorgram of spontaneous fusion of liposomes was monitored until the sensorgram readings begin to level out. This repeatedly occurred at the response level that is in the order of 7000–8000 RU above the original baseline.

The Cry4Ba-DIII truncate or its 65-kDa full-length protein at different concentrations was injected onto the resulting membrane-coated SPR surface at flow rate of 50 μL/min for 3 min. Subsequently, the sensor chip bound with the proteins was washed with running buffer (50 mM carbonate buffer, pH 9.0) for 5 min to allow protein dissociation. At the end of each measurement, the chip surface was regenerated by three 1-min injections of 40 mM OG and subsequently equilibrated with the running buffer. When the baseline of the sensorgram returned to the preceding level of lipophilic dextran coated on the chip, a new tethered lipid bilayer was created by the injection of liposomes for the next binding experiment. Binding kinetic parameters were determined by fitting the binding curves of various protein concentrations simultaneously to the available binding models using BIAevaluation 3.2 (Biacore AB, Uppsala, Sweden). Prior to such fitting, response curves were prepared by subtracting with the signal generated simultaneously on the reference flow cell and normalizing the start of all curves to zero.

### 3.5. Proteinase K Protection Assays of the Cry4Ba Proteins

To assess the extent of insertion into lipid bilayers of the bound proteins, a protease-digestion protection assay was performed. After the dissociation step of each SPR experiment, 0.2 μg/μL of proteinase K (a non-specific protease) in 50 mM carbonate buffer, pH 9.0 (running buffer) was injected at flow rate of 5 μL/min for 10 min over the chip surface. Bovine serum albumin immobilized on the lipid membrane surface of L1 sensor chip (Biacore AB, Uppsala, Sweden) was used as a control.

### 3.6. Membrane Perturbation Assays of the Cry4Ba Proteins

The membrane-perturbing activity of the purified Cry4Ba-DIII truncate was assessed in comparison with its full-length toxin by measuring the toxin-induced leakage of calcein fluorescence (self-quenching fluorescent dye) from the dye-loaded lipid vesicles as previously described [41]. Large unilamellar vesicles (LUVs, mean diameter of 50–100 nm) encapsulated with 50 mM calcein were prepared from a lipid mixture (Avanti Polar Lipids, Inc., Alabaster, AL, USA) of PE, PC and Ch (10:10:1, *w*/*w*/*w*) by the extrusion method according to standard procedures [42].

The release of entrapped calcein was monitored as function of time *t* by measuring an increase in fluorescence emission at 520 nm (excitation at 485 nm) on a spectrofluorometer at 25 °C. Calcein efflux activity was normalized to fluorescence recovery *F_t_* defined as *F_t_* = (*I_t_* − *I*_0_)/(*I*_max_ − *I*_0_) × 100, where *I*_0_ is initial fluorescence intensity, *I*_max_ is the total fluorescence intensity detected upon addition of Triton X-100 (giving 100% efflux) and *I*_t_ is the fluorescence intensity detected at time *t* after adding the tested proteins.

### 3.7. Single-Channel Analysis of the Cry4Ba Proteins via Planar Lipid Bilayers (PLBs)

Experimental procedures used to perform single channel analysis in PLBs were described previously [43], with some modifications. A lipid mixture of PE, PC and Ch (7:2:1, *w*/*w/w*) dissolved in decane was painted on a 200-μm aperture in a 1 mL-Delrin cup. Toxin samples (~1 μg/mL; prepared in 50 mM carbonate buffer, pH 9.0) were added into the *cis* compartment containing recording buffer (150 mM KCl, 10 mM Tris, pH 9.0 and 1 mM CaCl_2_). Single-channel currents were recorded with Axopatch-1D amplifier (Axon Instruments, Union City, CA, USA) and signals (filtered at 10 kHz) were digitized with Digidata 1200 analog-to-digital converter (Axon Instruments, Union City, CA, USA) using Axoscope 8.0 software at a 50-kHz sampling frequency. Single-channel activity was specified by clearly resolved current (I) jumps in response to voltage (V) steps.

## Figures and Tables

**Table 1:** Association and dissociation rate constants (*k*_a_ and *k*_d_) obtained from fitting the SPR membrane-binding curves of individual Cry4Ba proteins to the two-stage reaction model.

Proteins	*k*_a_(M^−1^ s^−1^) ^a^		*k*_d_ (s^−1^) ^a^	
21-kDa DIII truncate	*k*_a1_ = 6.9 × 10^3^	[± 0.8 × 10^3^]	*k*_d1_ = 3.5 × 10^−2^	[± 0.5 × 10^−2^]
	*k*_a2_ = 1.5 × 10^−2^	[± 0.2 × 10^−2^]	*k*_d2_ = 5.1 × 10^−4^	[± 0.9 × 10^−4^]
65-kDa full-length toxin	*k*_a1_ = 1.9 × 10^4^	[± 1.1 × 10^4^]	*k*_d1_ = 7.6 × 10^−2^	[± 2.1 × 10^−2^]
	*k*_a2_ = 4.7 × 10^−3^	[± 0.6 × 10^−3^]	*k*_d2_ = 7.7 × 10^−4^	[± 1.5 × 10^−4^]

^a^ Values represent the mean of at least two independent experiments.

## Supplementary Materials

The following is available online at http://www.mdpi.com/2072-6651/11/2/62/s1, Figure S1: schematic diagram of immobilized bilayer membrane sensor chip surface.

## Abbreviations

ATR-FTIR	attenuated total reflection Fourier transform infrared
Cry	crystal
FPLC	fast protein liquid chromatography
LUVs	large unilamellar vesicles
OG	*n*-octyl-β-d-glucopyranoside
PLBs	planar lipid bilayers
SPR	surface plasmon resonance
