# Supplementary material for: The C-Terminal Domain of the Bacillus thuringiensis Cry4Ba Mosquito-Specific Toxin Serves as a Potential Membrane Anchor
Source: Toxins (Basel). 2019 Jan 23;11(2):62. doi: 10.3390/toxins11020062 (PMC6410236; doi:10.3390/toxins11020062)
Supplement: Supplementary file 1 [file toxins-11-00062-s001.pdf]

Supplementary Materials: The C-terminal Domain of the *Bacillus thuringiensis* Cry4Ba Mosquito-Specific Toxin Serves as a Potential Membrane Anchor

Anon Thammasittirong, Chompounoot Imtong, Wilaiwan Sriwimol, Somsri Sakdee and Chanan Angsuthanasombat

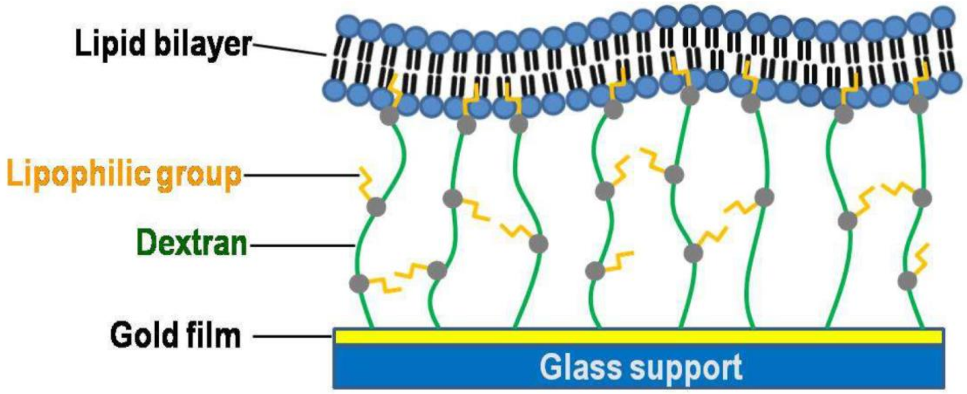

Figure S1. Schematic diagram of immobilized bilayer membrane sensor chip surface. After the binding to the sensor chip coated with carboxymethylated (lipophilic group) dextran, the immobilized liposomes would spontaneously fuse to form a large single-bilayer membrane.
